# Neuron-Glia Interaction as a Possible Glue to Translate the Mind-Brain Gap: A Novel Multi-Dimensional Approach Toward Psychology and Psychiatry

**DOI:** 10.3389/fpsyt.2013.00139

**Published:** 2013-10-21

**Authors:** Takahiro A. Kato, Motoki Watabe, Shigenobu Kanba

**Affiliations:** ^1^Department of Neuropsychiatry, Graduate School of Medical Sciences, Kyushu University, Fukuoka, Japan; ^2^Innovation Center for Medical Redox Navigation, Kyushu University, Fukuoka, Japan; ^3^Department of Management, School of Business, Monash University, Sunway, Malaysia

**Keywords:** translational research, neuron-glia interaction, mind-brain gap, unconscious, neuropsychoanalysis

## Abstract

Neurons and synapses have long been the dominant focus of neuroscience, thus the pathophysiology of psychiatric disorders has come to be understood within the neuronal doctrine. However, the majority of cells in the brain are not neurons but glial cells including astrocytes, oligodendrocytes, and microglia. Traditionally, neuroscientists regarded glial functions as simply providing physical support and maintenance for neurons. Thus, in this limited role glia had been long ignored. Recently, glial functions have been gradually investigated, and increasing evidence has suggested that glial cells perform important roles in various brain functions. Digging up the glial functions and further understanding of these crucial cells, and the interaction between neurons and glia may shed new light on clarifying many unknown aspects including the mind-brain gap, and conscious-unconscious relationships. We briefly review the current situation of glial research in the field, and propose a novel translational research with a multi-dimensional model, combining various experimental approaches such as animal studies, *in vitro* & *in vivo* neuron-glia studies, a variety of human brain imaging investigations, and psychometric assessments.

## Introduction

Neurons and synapses have long been the dominant focus of neuroscience, thus the pathophysiology of psychiatric disorders has come to be understood within the neuronal doctrine. However, the majority of cells in the brain are not neurons but glial cells including astrocytes, oligodendrocytes, and microglia. Traditionally, neuroscientists regarded glial functions as simply providing physical support and maintenance for neurons. Thus, in this limited role glia had been long ignored (1). Recently, glial functions have been gradually investigated, and increasing evidence has suggested that glial cells perform important roles in various brain functions. Digging up the glial functions and further understanding of these crucial cells, and the interaction between neurons and glia may shed new light on clarifying many unknown aspects including the mind-brain gap, and conscious-unconscious relationships. In addition, glial pathophysiology may explain the possible implications for the pathogenesis of major psychiatric disorders. The complexity of these aspects has yet to be well investigated. To explore these physiological and pathological aspects, novel translational methods should be applied with a multi-dimensional approach. Herein, we will briefly review the current situation of glial research in the field, and propose a novel translational research with a multi-dimensional model, combining various experimental approaches such as animal studies, *in vitro* & *in vivo* neuron-glia studies, a variety of human brain imaging investigations, and psychological/psychiatric assessments.

## Glial Roles and Pathology in Psychiatric Disorders

Recent biological studies have been revealing the important roles of glial cells in the process of neuropsychiatric disorders.

### Astrocytes

Astrocytes are the most prevalent cell type in human brain and contribute to the homeostasis of the brain by regulation of neuronal metabolism, modulation of CNS inflammation, and direct/indirect synaptic transmission such as MNDA receptors (2, 3). Astrocyte dysfunction has been critical for various neurological disorders (4). Recent studies have shown abnormal expression of glial fibrillary acid protein (GFAP) – a prototypical marker of astrocyte – in postmortem brain of patients with schizophrenia and major affective disorders (5–7). In addition, recent rodent studies have suggested that astrocytes modulate anxious and depressive behaviors (8, 9). On the other hand, direct modulating effects of antidepressants have also been revealed (10–13). Thus, astrocytes have been supposed to be a novel therapeutic target against various psychiatric disorders such as major affective disorders and bipolar disorders (14, 15).

### Oligodendrocytes

Oligodendrocytes contribute to brain development and homeostasis in the brain by formulating myelin around axons, supporting neuronal networks in the brain. Recently, novel oligodendrocyte functions have been revealed such as monitoring neuronal activities via myelin-forming oligodendrocytes (16) and modulating the conduction velocity of action potentials along axons in the rat hippocampus (17). Dysfunctions of oligodendrocytes have been indicated in psychiatric disorders, especially schizophrenia and major affective disorders, from a series of genetic studies (18, 19), postmortem studies (20–22), and diffusion tensor imaging (DTI) studies (23–27). A novel animal model of schizophrenia has been developed by treating a copper chelator, which induces oligodendrocyte dysfunction and white matter abnormality as demyelination and schizophrenia-related behaviors (28, 29). Cuprizone caused marked behavioral changes (working memory deficit) indicated by the results of Y-maze task, which showed an increase in the number of arm entries and a decrease in alternation behavior. These cuprizone-induced behavioral changes were effectively prevented by chronic administration of quetiapine, an atypical antipsychotic, which also diminished demyelination (28). On the other hand, recent rodent studies have revealed the interaction between oligodendrocyte dysfunction and social behaviors. Makinodan et al. reported that oligodendrocyte dysfunction is formed by early-period social isolation and this maladaptive environment induces working memory deficit associated with prefrontal cortex (PFC) function in later life (30). Liu et al. reported that protracted social isolation of adult mice induces behavioral, transcriptional, and ultrastructural changes in oligodendrocytes of the PFC and impairs adult myelination (31).

### Microglia

Microglia are unique glial cells of mesodermal origin in the brain that act as “brain macrophage”; immunological/inflammatory players by moving around and releasing cytokines and free radicals (32, 33). Thus, microglia have proved to play important roles in various brain pathologies such as neurodegenerative diseases and neuropathic pain via inducing inflammation and oxidative stress (34–36). Recently, microglia have been revealed to have direct contact with synapses and have proved to play crucial roles in neuronal development through synaptic pruning (37–39). Postmortem studies have shown microglial activation in the brain of patients with schizophrenia and major affective disorders, especially suicide victims (40–42). In addition, positron emission tomography (PET) imaging studies using the peripheral benzodiazepine receptor bindings has shown that microglia are activated in patients with schizophrenia (43–45) and autism (46). On the other hand, minocycline, an antibiotic with inhibitory effects on microglial cells, has been reported to have therapeutic effects on schizophrenia and unipolar psychotic depression (47–49). In addition, rodent *in vitro* studies have proved the novel effect of psychotropic drugs (atypical antipsychotics such as risperidone and aripiprazole, and antidepressants such as paroxetine and sertraline, both selective serotonin reuptake inhibitors) directly on microglia by suppressing release of inflammatory cytokines and free radicals (50–54). Thus, microglia are suggested to play key roles in psychiatric disorders (53, 55, 56).

In the brain, neurons, astrocytes, oligodendrocytes, and microglia are mutually communicating with each other, by direct-contacting or via neurotransmitters and other various small molecules (57), and dysfunction of neuron-glia communication may induce pathological conditions not only in neurodegenerative diseases (58) but also in psychiatric conditions such as psychosis, depression, and anxiety. The above-mentioned recent findings strongly suggest that glial cells contribute to psychiatric disorders, while the underlying mechanisms have not been clarified.

## Possible Glial Roles in Human Mental Functions

Until recently, the actual roles of glia in mental activities, especially for healthy humans, have not been investigated. As the first step to clarify this unexplored field, we have started to conduct a series of social decision-making experiments with healthy human subjects using minocycline, a microglial inhibitor (59–61). Healthy Japanese adult males made a monetary decision about whether or not to trust an anonymous partner after a 4-day oral administration of minocycline. Our first trial revealed that the minocycline group showed a positive correlation between their monetary score in trust game and their evaluation scores of others’ trustworthiness in a questionnaire (Yamagishi’s General Trust Scale), but surprisingly the placebo group did not (60). It would be rational to consider the monetary and questionnaire score to be positively correlated because both scores measure the other’s trustworthiness, but there was no positive correlation with the placebo group. The questionnaire is measuring only conscious-level decision-making, on the other hand the monetary score is measuring the final decision-making affected by not only the conscious but also the unconscious; suggesting that some unconscious noisy factors seem to be affecting the placebo group. Treatment with minocycline, a microglial inhibitor, has shown the positive correlation. Therefore, this first trial has indicated that microglial activation may cause “unconscious noises” against appropriate social decision-making, and inhibiting microglial activity may reduce such noise (60). In a next trial with larger samples, we additionally measured the effects of anxiety and personality as candidates for “noise” factors, by using Temperament and Character Inventory (TCI) and State-Trait Anxiety Inventory (STAI) (59). The monetary score in trust game was significantly lower in the minocycline group. Interestingly, participants’ ways of decision-making were significantly shifted; certain personality traits (cooperativeness, reward dependence, and self-directedness) proved to be the main modulating factors of decision-making in the placebo group, on the other hand the minocycline group was mainly modulated by state anxiety and trustworthiness. Our results of the second trial suggest that minocycline led to more situation-oriented decision-making, possibly by suppressing the effects of personality traits, and furthermore that personality and social behaviors might be modulated by microglia. Interestingly, cooperativeness has proved to be the most influential factor in the process of decision-making in the placebo group of Japanese participants (59). It is widely known that cooperativeness and cooperative behaviors have been highly respected and emphasized aspects in Japanese society. Thus, of course, these aspects are ingrained during childhood by various sociocultural experiences within family relationships, schools, and other areas of society in Japan. Early-life events may activate human microglia, establish a certain neurosynaptic connection, and this formation may determine personality and personality-oriented social behaviors in later life (59, 62). If these experiments are conducted in other countries with different sociocultural backgrounds, other personality traits may be identified.

**Figure 1:**
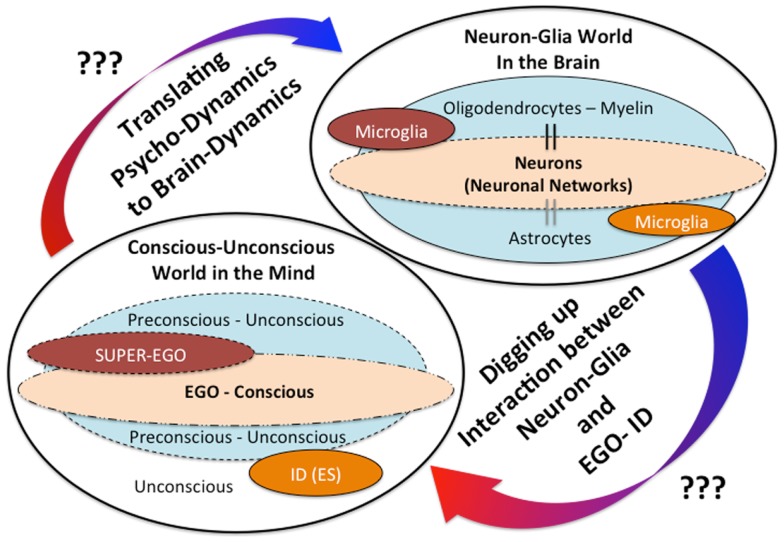
**The mind-brain gap from a novel glial neuropsychoanalytic perspective**. The interaction between the mind and the brain has not been well understood. Freud, the founder of psychoanalysis, proposed the conception of mind structure models consisting of the following three components: *the ID* (unconscious/instinctual drives), *the EGO* (the exclusive apparatus of the conscious mind), and *the SUPER-EGO* (which represses *the id* in order to avoid any disruptions of rational thought). The existence and the significances of these mental components may be explained by future understandings of the neuron-glia interactions. The hypothetical interaction between the ID (unconscious drives) and microglia has already been proposed in our recent theoretical paper (62).

In addition, we have recently reported a possible outcome that minocycline, a microglial inhibitor, also reduces the risk of the “honey trap” during economic exchanges between males × females (61). Males tend to cooperate with physically attractive females without careful evaluation of their trustworthiness. In our experiment, young healthy male participants made risky choices (whether or not to trust female partners, identified only by photograph, who had decided in advance to exploit the male participants). The results show that trusting behavior in male participants significantly increased in relation to the perceived attractiveness of the female partner, but attractiveness did not impact trusting behavior in the minocycline group (61). These novel effects of minocycline may highlight the unknown roles microglia play in deeper human mental activities; microglia may modulate our unconscious drives in various social settings. The above-mentioned findings shed new light on the dark side of microglial social/mental functions in humans, especially highlighting the role of microglia for the unconscious. In the same way that Sigmund Freud, the founder of psychoanalysis, proposed that our behaviors must be controlled by the unconscious world, microglia may unconsciously control our behaviors. How do microglia act as fundamental mediators between the conscious and the unconscious world? What do neurobiological mechanisms justify their eventual role in bridging the gap between neuroscience and psychoanalysis? Answers to the above questions are not yet clear, but we have recently proposed a hypothesis creating a link between Freud’s unconscious drives such as the death drive and microglial activation (62). For example, microglial maladaptive over-activation in a certain brain region may activate human aggressive behaviors as a result of destructive drives [For the details, please see our recent article; Ref. (62)]. In the brain, not only microglia but also other glia such as astrocytes and oligodendrocytes exist, thus complicated neuron-glia interactions may modulate our mental activities including the unconscious (Figure 1). Further research should be applied to clarify these unresolved questions.

After Freud’s theory of unconscious roles in behaviors which was initially identified in the 1980s (63), Pribram and his colleagues have developed this theory in terms of a better articulated model of neural computation (64, 65). In addition, recent neuropsychoanalytic movements have been updating Freud’s theory with modern sophisticated methods of cognitive neuroscience (66–72). Thus, these recent approaches have been revealing the underlying mechanisms of implicit processing in a variety of information-processes including the social processes using rodent experiments. At present, the link underlying mechanisms between neuron-glia interactions and the conscious-unconscious relationship is largely unsolved, and few experimental methods have been developed to test these unknown brain mechanisms at either the microscopic or macroscopic level. Unconscious processing needs to be given a greater focus in terms of brain mechanisms. One possible solution is the novel ontogenetic approach; called “optogenetics” (73–76). Optogenetics is a revolutionary technique involving taking a light-activated gene (called a channel rhodopsin) targeted into a single neuron type. This technique enables to clarify direct interaction between activation of specific neuron in specific region by light and the resulting outcomes such as behaviors and emotional reactions at rodent level. A recent study has interestingly shown that activation of specific neurons in hippocampus produce a false memory in mice (77). Further technological developments in modulating glial cells by light and in activating both neurons and glial cells at the same time, by multiple fluorescent lights, may shed new light on resolving unknown roles of glia and neuron-glia interaction in behaviors and the conscious-unconscious. Functional roles and pathological contributions of astrocyte, oligodendrocyte, and/or microglia in conscious or unconscious processes have not been well understood, and we hypothesize that each cell may differently contribute to these physical and/or pathological processes in different brain regions such at the brainstem, limbic, or thalamocortical region, respectively. Future developments in optogenetics may clarify these unknown aspects.

## Limitation and Future Perspectives of Neuro-Glia Research on Psychology and Psychiatry

To explore the above-mentioned hypothesis, further translational research is needed. Several limitations should be made note of at the present stage. At first, rodent studies focusing on the unconscious are limiting. Even if the unconscious exists in rodents, it seems to be impossible to measure the unconscious in rodents devoid of human language capabilities. Therefore, to uncover the unconscious mechanisms, we have no alternative method except examining actual human subjects. We have no specific drugs to modulate glial cells utilized in human, and minocycline is reported to have other brain functions in addition to microglial inhibition (78, 79). On the other hand, some brain imaging techniques enable us to explore the unknown roles of glial cells such as DTI technique and PET imaging using the peripheral benzodiazepine receptor bindings, while the specificities of these imaging methods are not at satisfactory levels (80). On the other hand, we can reconsider previous findings of brain imaging experiments. Functional MRI (fMRI) is a brain imaging procedure measuring brain activity by detecting associated changes in blood flow (81, 82). Outcomes of fMRI have long been believed to monitor solely neuronal activities, because cerebral blood flow and neuronal activation have been thought to be almost equivalent. However, not only neuronal activities but also glial activities, especially astrocyte activities, rely on cerebral blood flow. Therefore, at least to some extent, brain activities expressed by fMRI may be showing a part of glial activation. In addition, MR spectroscopy (MRS) is one of the novel imaging approaches to measure dynamic brain functions focusing on metabolomics including glia-related molecules. For example, myo-inositol, which can be measured by MRS, is regarded as a marker of astrocyte activity (83). These imaging methods and combination of these imaging techniques may shed new light on clarifying unknown roles of glia in psychiatric disorders (84, 85). For example, activated microglia-derived myelin damage has been indicated in the pathophysiology of schizophrenia by rodent experimental models (28, 29, 86, 87), while it is not confirmed in human subjects. Combination of human DTI and PET may clarify the mutual interaction between microglial activation and myelin damage in schizophrenia patients. On the other hand, connectivity of each brain region has been important in the understanding of the roles of brain functions from the era of Hughlings Jackson. fMRI studies have revealed the importance of these aspects (88, 89), and the recent development of DTI is showing us the significance of more complicated brain networks focusing on not only neurons but also glial cells such as oligodendrocytes (90, 91).

**Figure 2:**
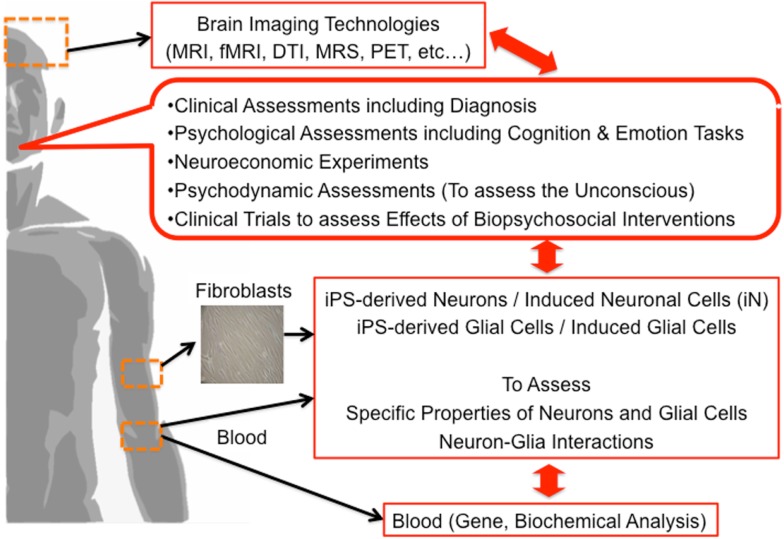
**A novel multi-dimensional approach toward psychology & psychiatry**.

Finally, we propose the multi-dimensional approach to clarify the underlying brain mechanisms of mental functions including the unconscious (Figure 2). Based on our discussion, we believe that not only neurons but also glial cells have a vital role in the process of mental activities, a novel approach focusing on neuron-glia interactions should be applied. Combination of brain imaging techniques focusing on both neurons and glial cells should be applied (24, 26, 27, 43–46, 92–94). The most significant limitation in human brain research is that we cannot obtain living brain cells, including glial cells, from living human subjects from an ethical perspective. Presently, we can apply an alternative method; human brain cells such as neuronal cells can be established from somatic cells (not from the brain) such as skin fibroblasts by utilizing the gene-modification technique of induced pluripotent stem (iPS) cells. In addition, recently, neuronal cells are more easily established from directly conversion of human skin fibroblasts, called induced neuronal (iN) cells (95–99). Novel methods of establishing glial cells are strongly warranted based on iPS or direct conversion techniques in the near future. Multi-dimensional aspects of same human subjects, from genes, blood, brain imaging, psychometrics, social function, unconscious functions, psychodynamic assessments to molecular functions of somatic tissue-derived neuronal and glial cells, should be investigated and analyzed together (Figure 2). This approach may explore the novel roles of glial cells in various human mental activities including the unconscious. The application of this method for psychiatric patients should also be encouraged in the establishment of novel diagnostic methods and novel therapies.

## Conflict of Interest Statement

The authors declare that the research was conducted in the absence of any commercial or financial relationships that could be construed as a potential conflict of interest.
